# Butanol Purified Food Allergy Herbal Formula-2 Has an Immunomodulating Effect *ex-vivo* in Pediatric Crohn's Disease Subjects

**DOI:** 10.3389/fmed.2021.782859

**Published:** 2021-11-29

**Authors:** Xin Chen, Joanne Lai, Ying Song, Nan Yang, Sacha Gnjatic, Virginia Gillespie, William Hahn, Ezra Chefitz, Nanci Pittman, Jacqueline Jossen, Keith Benkov, Marla Dubinsky, Xiu-Min Li, David Dunkin

**Affiliations:** ^1^Division of Pediatric Gastroenterology, The Icahn School of Medicine at Mount Sinai (ISMMS), New York, NY, United States; ^2^The Mindich Child Health and Development Institute (MCHDI), The Icahn School of Medicine at Mount Sinai (ISMMS), New York, NY, United States; ^3^Academy of Chinese Medicine, Henan University of Chinese Medicine, Zhengzhou, China; ^4^Microbiology and Immunology, New York Medical College, Valhalla, NY, United States; ^5^General Nutraceutical Technology, Elmsford, NY, United States; ^6^Division of Hematology and Medical Oncology, New York, NY, United States; ^7^The Precision Immunology Institute, The Icahn School of Medicine at Mount Sinai (ISMMS), New York, NY, United States; ^8^Department of Comparative Pathology, The Icahn School of Medicine at Mount Sinai (ISMMS), New York, NY, United States

**Keywords:** Crohn's disease, herbal therapy, pre-clinical drug testing, immunomodulatory, drug development

## Abstract

**Background:** TNF-α has a major role in the pathogenesis of Crohn's disease (CD). In contrast, GM-CSF may be beneficial for its anti-inflammatory role in a subset of patients with CD with antibodies against GM-CSF as seen in prior trials of GM-CSF which resulted in clinical improvement in CD. We developed butanol purified Food Allergy Herbal Formula-2 (B-FAHF-2) by refining FAHF-2. FAHF-2 suppressed TNF-α production by human peripheral blood mononuclear cells (PBMCs) and colonic mucosa, and abrogated colitis in a murine model. We sought to examine the effect of B-FAHF-2 and the herbs that comprise it on TNF-α and GM-CSF production as a potential herbal therapy for the treatment of CD.

**Methods:** B-FAHF-2 was examined using high pressure liquid chromatography (HPLC) and compared to the original formulation, FAHF-2. PBMCs from pediatric patients with CD were cultured with lipopolysaccharide and B-FAHF-2, individual herbs or medium alone. Colonic biopsy specimens were cultured with or without B-FAHF-2. TNF-α and GM-CSF were measured by enzyme-linked immunosorbent assay (ELISA). B-FAHF-2 efficacy was tested *in vivo* in the CD45Rb^hi^ transfer model.

**Results:** B-FAHF-2 had a similar HPLC fingerprint as FAHF-2 but decreased TNF-α production by PBMCs and colonic mucosa from pediatric CD subjects at 20% of the FAHF-2 dose. B-FAHF-2 increased GM-CSF production by PBMCs and colonic mucosa from pediatric CD subjects including those with antibodies to GM-CSF. Of B-FAHF-2's herbal constituents, only Huang Bai suppressed TNF-α and increased GM-CSF production. In the murine model, B-FAHF-2 treatment alleviated colitis.

**Conclusions:** B-FAHF-2 decreased TNF-α production by PBMCs and colonic mucosa from pediatric subjects at a lower dose than FAHF-2. B-FAHF-2 also increased GM-CSF production by PBMCs independent of antibodies. B-FAHF-2 may have a benefit in CD patients.

## Introduction

Crohn's disease (CD), a form of inflammatory bowel disease (IBD), is a life-long disease characterized by chronic and relapsing inflammation of the gastrointestinal tract. The prevalence of CD varies based on region, race, age, and environment. The annual incidence of CD ranges from 16.7 to 318.5 per 100,000 in North American, 0.6 to 322 per 100,000 in Europe, and 0.88 to 67.9 per 100,000 in Asia and the Middle East (1). Among CD patients, 20–25% are diagnosed before 17 years old. The incidence of pediatric CD is steadily increasing (2). CD has multiple clinical phenotypes and disease severities that determine which therapy is utilized. Currently, there are numerous treatment options for children and adults with moderate-to-severe CD, but few that are approved to treat those with mild-to-moderate disease. The only FDA approved medication for treatment of mild-to-moderate CD in children 8 years and older is Entocort EC (budesonide). The indications allow for up to 10 weeks of use in active disease but not for use as a maintenance therapy in children. Budesonide is a steroid compound and as such may have the same side effects as corticosteroids. Other medications including immunomodulators and biologics are used off-label for mild-to-moderate CD in children and have risks for significant adverse effects. Thus, there is a need for the development of new therapies.

Herbal therapies may fill this therapeutic void and there is a growing interest in complementary treatments (3). Food Allergy Herbal Formula-2 (FAHF-2) is composed of nine Chinese herbal medications that are originally part of the traditional Chinese herbal formula, Wu Mei Wan, that has been used safely to treat colitis in China and Japan for thousands of years (4). FAHF-2 has been shown in both murine and human studies of food allergy to inhibit both adaptive and innate immune pro-inflammatory cytokine responses in peripheral blood mononuclear cells (PBMCs) and *in vivo* (5–10). Interestingly, in food allergic subjects, FAHF-2 induced potent suppression of TNF-α, one of the major inflammatory cytokines involved in the pathogenesis of IBD. Anti-TNF antibody-based biologics are one of the most efficacious groups of medications currently in use to treat IBD. That led to our investigation on the effects of FAHF-2 in IBD. We showed that FAHF-2 suppresses TNF-α, IFN-γ, IL-1β, IL-6, IL-12 and IL-17 production from both PBMCs and inflamed colonic mucosa from pediatric subjects with CD, and abrogates murine colitis in the CD45RB^hi^ transfer model (5). FAHF-2 affects immune responses by disrupting the NF-kB pathway (5).

FAHF-2's effects are immunomodulatory and not globally immunosuppressive as evidenced by the effect FAHF-2 has on the production of IFN-γ in different inflammatory diseases. In food allergy, FAHF-2 stimulates increased production of IFN-γ (8, 11), whereas in CD it decreases IFN-γ production (5). In addition, FAHF-2's effects on chemokines and growth factors shown here for the first time, demonstrate that FAHF-2 suppresses production of numerous chemokines (IP-10, MIP-1β, eotaxin, RANTES, MIG, MCP-1, and MIP-1α) and stimulates production of granulocyte-macrophage colony-stimulating factor (GM-CSF) by PBMCs from pediatric subjects with CD.

GM-CSF in particular plays an integral role in intestinal innate immunity, immune tolerance and the induction of intestinal regulatory T cells (12, 13). Pediatric and adult CD patients with ileal and stricturing or penetrating disease were shown to have higher levels of GM-CSF autoantibodies (14). A recombinant human form of GM-CSF, sargramostim, had positive effects in a small pilot study of CD patients. A subsequent larger placebo-controlled trial showed that GM-CSF therapy decreased disease severity and induced mucosal healing, but failed to demonstrate a clinical response (15). The larger study failed to target subjects with deficiencies or antibodies against GM-CSF and this may have been the reason for its failure. Heterogeneity in pathogenic mechanisms exists. Thus, an increase in GM-CSF may be therapeutic for a subgroup of CD patients, and we therefore continued to investigate the ability of FAHF-2 and its derivatives to alter GM-CSF.

FAHF-2 shows promise as a possible therapy for CD. Clinical trials of FAHF-2 in food allergy however, revealed that the high burden of pills needed in clinical trials contributed to non-compliance (16). Our goal was to lower the pill burden needed to use FAHF-2 in treatment protocols. We therefore developed butanol purified FAHF-2 (B-FAHF-2) which could be used at a fraction of the dose. We also tested the individual herbs that comprise FAHF-2 and B-FAHF-2 for their effects on TNF-α and GM-CSF as representative of their ability to modulate the immune response in CD. We found that the purified form, B-FAHF-2, significantly suppressed the production of TNF-α by PBMCs and intestinal mucosal from pediatric subjects with CD and abrogated colitis in a murine model at a much lower dose than FAHF-2. B-FAHF-2 also increased production of GM-CSF by PBMCs and intestinal mucosa and this was also true in a small subset of our CD subjects with antibodies against GM-CSF. Consistent with traditional Chinese medicine practices, the individual herbs within FAHF-2 and B-FAHF-2 were not as effective as the whole in suppressing TNF-α and increasing GM-CSF.

## Materials and Methods

### FAHF-2 and B-FAHF-2 Production, Quality Control, and Dose Derivation

FAHF-2 and B-FAHF-2 were obtained from Xiyuan Chinese Medicine Research and Pharmaceutical Manufacturer, Chinese Academy of Chinese Medicine Sciences, Beijing, China, a good manufacturing practice (GMP) certified facility. Herbal components of FAHF-2 are listed in Table 1. All herbs were inspected for identity and quality by licensed pharmacists. All plant names have been checked with http://www.theplantlist.org except for *Ganoderma lucidum* which is a fungus and not listed there. Voucher specimens of the raw herbs were archived in the laboratory of Dr. Xiu-Min Li. The manufacturing process of FAHF2 is as follows: *Ganoderma lucidum* powder was generated by decoctions by boiling small pieces *Ganoderma lucidum* twice (2 h each time). *Panax ginseng* was extracted twice with 80% aqueous ethanol, then filtered, combined, and evaporated until there was no residual ethanol. The other 7 herbs were combined and boiled twice in water (1.5 h each time). The decoction was collected, combined, and purified by ethanol precipitation. *Panax ginseng* was mixed with the 7 herbs extract, dried into a powder, and then combined with the *Ganoderma lucidum* powder to form FAHF-2 (17, 18).

**Table 1:** The composition of FAHF-2.

**Latin name**	**Chinese name**	**Plant part**	**Occupied** **percent**
*Prunus mume*	Wu-Mei	Fruit	20%
*Zanthoxylum schinifolium*	Chuan-Jiao	Pericarp	2%
*Angelica sinensis*	Dang-Gui	Root	6%
*Zingiber officinalis*	Gan-Jiang	Root	6%
*Cinnamomum cassiae*	Gui-Zhi	Burgeon	4%
*Phellodendron chinense*	Huang-Bai	Cortex	4%
*Coptis chinensis*	Huang-Lian	Root	6%
*Panax ginseng*	Hong-Shen	Root	6%
*Ganoderma lucidum*	Ling-Zhi	Mushroom	46%

Extensive quality control, analytical chemistry data and batch consistency pertaining to the formula have been published previously (11). We identified chemical markers by high-pressure liquid chromatography (HPLC) and liquid chromatography (LC) mass spectrometry. Endotoxin levels were measured using the Pyrogent Plus assay kit (Lonza, MA) and were below 0.03 EU/ml, the limit of sensitivity for this kit.

Given the generally favorable safety profile, the dose ranges of most Chinese herbal formulations are wide. Initially, the dose used in our FAHF-2 studies was converted from the human daily dose of the Wu Mei Wan formula and Ling Zhi extract at a medium to high dose range using a conversion table of equivalent effective dose ratios from humans to animals based on body surface area (19). This dose was consistently effective in protecting peanut allergic mice from anaphylaxis, and at abrogating colitis in a murine model (5). B-FAHF-2 is 20% of FAHF-2 by weight because of the elimination of non-medicinal components and thus the dose used in our experiments is derived by using 20% of the dose of FAHF-2 (250 μg/ml). The effective dose (60 μg/ml) of B-FAHF-2 was found to be 20% that of FAHF-2 based upon cell culture assays and *in vivo* models of peanut allergy (6). For the individual herbs within FAHF-2 and B-FAHF-2, the doses used correspond to the equivalent dose found in the full compound. Equivalent doses were as follows: Wu Mei (WM) 50 μg/ml, Chuan Jiao (HJ) 125 μg/ml, Dang Gui (DJ) 125 μg/ml, Gan Jiang (GJ) 125 μg/ml, Gui Zhi (GZ) 125 μg/ml, Huang Bai (HB) 125 μg/ml, Huang Lian (HL) 125 μg/ml, Hong Shen (HS) 125 μg/ml, and Ling Zhi (LZ) 125 μg/ml.

### HPLC of FAHF-2, and B-FAHF-2

The instruments used were a Waters 2690 HPLC system coupled to a 2996 PDA detector (Waters, Milford, MA). B-FAHF-2 tablets were ground, and a 22.5 mg/mL solution was prepared using 1:1 ratio of mobile phase mixture. The B-FAHF-2 solution was centrifuged at 10,000 rpm for 10 minutes. 10μL of the supernatant was injected into the HPLC system and separated on a ZORBAX SB-C18 (4.6 × 150 mm, 5 μm) column (Agilent, Santa Clara, CA). The mobile phase A was made of 0.1% of formic acid aqueous solution. Mobile phase B was acetonitrile. The separation was performed at 1 min/mL flow rate following a linear gradient elution of 2–25% mobile phase B in 45 min, 25–35% B in the following 25 min, 35–55% in the next 15 min, 55–75% in another 10 min. This mobile phase composition was maintained for 5 min and then rapidly switched to 2% mobile phase B. An equivalent amount of FAHF-2 formula, 99 mg/mL, was also prepared following the same procedure as for B-FAHF-2. 10μL of FAHF-2 supernatant was analyzed on the HPLC system. Data was collected and processed with Waters' Empower software.

### Subjects

Human studies were approved by the Institutional Review Board of the Icahn School of Medicine at Mount Sinai (No. 11-00808). All participants provided written informed consent for participation in the study. Blood samples (*n* = 29) and inflamed colonic biopsy specimens (*n* = 20) were collected from newly diagnosed pediatric CD patients (8–19 years old) naïve to medications that could alter immune responses including: immune-modulators, biologics or steroids. CD patients were diagnosed based on standard clinical, radiographic and endoscopic criteria. Blood and colonic biopsy specimens were also collected from non-IBD control subjects (*n* = 12). Patient characteristics are outlined in Table 2.

**Table 2:** Subject characteristics based upon the Montreal classification for CD.

	**CD** **(***n*** = 29)**	**Non-IBD** **(***n*** = 12)**
Gender (male/female)	21/8	6/6
Age, mean ± SD	13 ± 3.2	15.8 ± 2.5
Location (L2/L3)	6/23	N/A
Behavior (B1/B2/B3)	26/0/3	N/A
Perianal (N/Y)	26/3	N/A

*L2, colonic; L3, ileocolonic; B1, non-stricturing, non-penetrating; B2, stricturing; B3, penetrating*.

### PBMC Separation, Cell Culture and Cytokine, Chemokine, and Growth Factor Measurements

PBMCs were isolated by Ficoll Hypaque (Pharmacia, Piscataway, NJ) with density gradient centrifugation. Purified PBMCs were cultured in medium containing 5% FBS with or without FAHF-2 (250 μg/ml), B-FAHF-2 (60 μg/ml), individual herbs in B-FAHF-2 (doses noted above) or dexamethasone (10^−3^ μM/ml, positive control) for 24 h followed by LPS (2 μg/ml) stimulation or no stimulation for an additional 24 h (8). Cytokine, chemokine and growth factor levels in the culture supernatants were determined by ELISA per the manufacturer's instructions (BD Biosciences), by multiplex immunoassay or by cytometric bead array. Multiplex assays were performed per the manufacturer's instructions (Luminex Human assay, Invitrogen, Grand Island, NY). Cytometric bead array was performed per the manufacturer's instructions (BD Biosciences).

### Biopsy Preparation and Culture

Colonic biopsies were washed with PBS and then cultured with or without B-FAHF-2 (60 μg/ml) in complete RPMI (10% FBS and GPS) with phosphatase and protease inhibitors overnight. The volume of culture medium was based upon the weight of each sample so that the size of the biopsies could be standardized. Supernatants were filtered and TNF-α and GM-CSF were assessed by ELISA (BD Biosciences) per the manufacturer's instructions.

### GM-CSF Antibody Measurement

Serum was tested by ELISA. Briefly, samples were diluted serially in 4-fold increments from 1/100 to 1/25,000 and assayed for the presence of IgG and IgA against GM-CSF (Sargramostim, Genzyme), as well as irrelevant control recombinant proteins. Each plate included positive control sera from patients with pulmonary alveolar proteinosis as well as a negative control healthy donor serum pool, expected, respectively, to react or not with GM-CSF.

### CD45RB^hi^ T Cell Transfer Model of Colitis

Animal studies were approved by the Institutional Animal Care and Use Committee at Mount Sinai. Cells were obtained from C57BL/6 wild type mice and enriched using an EasySep Cell Isolation Kit (STEMCELL Technologies Inc., Vancouver, CA) that depletes CD8^+^, CD11b^+^, CD11c^+^, CD19^+^, and B220^+^ cells by negative selection. The resulting CD4^+^ enriched population was labeled with FITC-conjugated anti-CD4 Ab, APC-conjugated anti-CD62L Ab and PE-conjugated anti-CD45RB Ab (eBiosciences). Subpopulations of CD4^+^ cells were sorted by flow cytometry. CD4^+^CD45RBhi T cells (3.5 x 10^5^) were adoptively transferred by intraperitoneal injection into recipient RAG1^−/−^ mice. Transfer was confirmed by flow cytometry of peripheral blood in all mice after 2–4 weeks. To prevent the progression to colitis, the recipient mice were fed B-FAHF-2 (50 mg/day) daily by gavage 24 h after CD4^+^CD45RBhi T cells transfer. Control mice received water by gavage. Weights were recorded semi-weekly. All mice were sacrificed once any mouse lost 20% of their initial weight. Colonic histology was scored for inflammatory infiltrates and epithelial damage by a pathologist blinded to the treatment group (20, 21). A total score with a maximum of 20 was determined by the summation of the following sub-scores: mucosal involvement: 0 = normal, 1 = 3–10 mucosal neutrophils/hpf, 2 = more than 10 mucosal neutrophils or rare crypt abscesses, 3 = multiple crypt abscesses or erosion; submucosal involvement: 0 = normal,1 = focal aggregates of neutrophils, 2 = neutrophil infiltration with expansion of submucosa, 3 = diffuse neutrophil infiltration; muscularis: 0 = normal, 1 = scattered neutrophils within the muscularis, 2 = neutrophil infiltration with focal effacement of the muscularis, 3 = extensive neutrophil infiltration with transmural effacement of the muscularis; crypt damage: 0 = normal, 1 = loss of the basal one third, 2 = loss of the basal two thirds, 3 = entire crypt loss, 4 = crypt loss with surface erosion, 5 = confluent, extensive erosion; ulcerations: 0 = absence of ulceration, 1 = one to two ulcer foci, 2 = three to four ulcer foci, 3 = confluent extensive ulceration. Colonic samples were cultured overnight in medium containing phosphatase and protease inhibitors. Cytokine secretion was measured in the supernatants (IL-4, IL-6, IL-10, IL-12, TNF-β, IFN-γ, IL-17A) by cytometric bead array (BD Biosciences) and ELISA for GM-CSF (BD Biosciences) per the manufacturer's instructions. RT-PCR was performed to look at regulatory elements within the colonic mucosa including TGFβ1, TGFβ2, Foxp3, and IL-10. Colon samples' RNA extraction and complementary DNA (cDNA) transcription were performed as previously described. (22) SYBR™ Green Master Mix (Thermo Fisher Scientific, Fair lawn, NJ) was used to perform RT-PCR. The target gene mRNA expression was normalized to the untreated group and calculated with the _ΔΔ_CT method. The primer sequences are listed in Table 3. Immunofluorescence (IF) staining of mouse colon sections was performed according to the previously described protocol with slight modifications (23). Paraffin embedded slides were dewaxed by serial xylene, xylene/ethanol (1:1), 100% ethanol, 95% ethanol, 70% ethanol, and H_2_O. Then slides were unmasked in heat antigen retrieval solution in the microwave. After permeabilization for 10 min at room temperature (RT), the slides were blocked with 20% goat serum in PBS for 1 h at room temperature, and then incubated with CD4-FITC and F4/80-APC (eBiosciences, San Diego, CA) overnight at 4°C. DAPI was used to stain cell DNA.

**Table 3:** Real-time PCR primer sequences.

**Target**	**Forward sequences (5^′^-3^′^)**	**Reverse sequences (5^′^-3^′^)**
TGF-β1	CCCGAAGCGGACTACTATGC	CGAATGTCTGACGTATTGAAGAACA
TGF-β2	CACCCAGCGCTACATCGATAG	CAGCGTCTGTCACGTCGAA
IL-10	TTTGAATTCCCTGGGTGAGAA	GCTCCACTGCCTTGCTCTTATT
Foxp3	ACTGGGGTCTTCTCCCTCAA	CGTGGGAAGGTGCAGAGTAG
Gapdh	GGTGGTCTCCTCTGACTTCAACA	GTTGCTGTAGCCAAATTCGTTGT

### Statistics

Paired *T*-tests with Wilcoxon signed-rank test were used when comparing the same samples without and with treatment. Comparisons between multiple groups were done using one-way ANOVA. This was followed by either non-parametric Mann-Whitney U test or Bonferroni analysis when appropriate. Statistical differences between groups for the colitis model were determined by Mann-Whitney *T*-test assuming non-normally distributed data. Data analysis was done using Prism software (GraphPad, San Diego, CA). A value of *p* < 0.05 was considered statistically significant. *P*-values are indicated by ^*^p < 0.05, ^**^p < 0.01, ^***^p < 0.001, ^****^p < 0.0001.

## Results

### B-FAHF-2 Contained the Same Compounds as FAHF-2 and Suppressed TNF-α Production by PBMCs and Colonic Mucosa From Pediatric Subjects With CD at 20% of the Dose of FAHF-2

**Figure 1:**
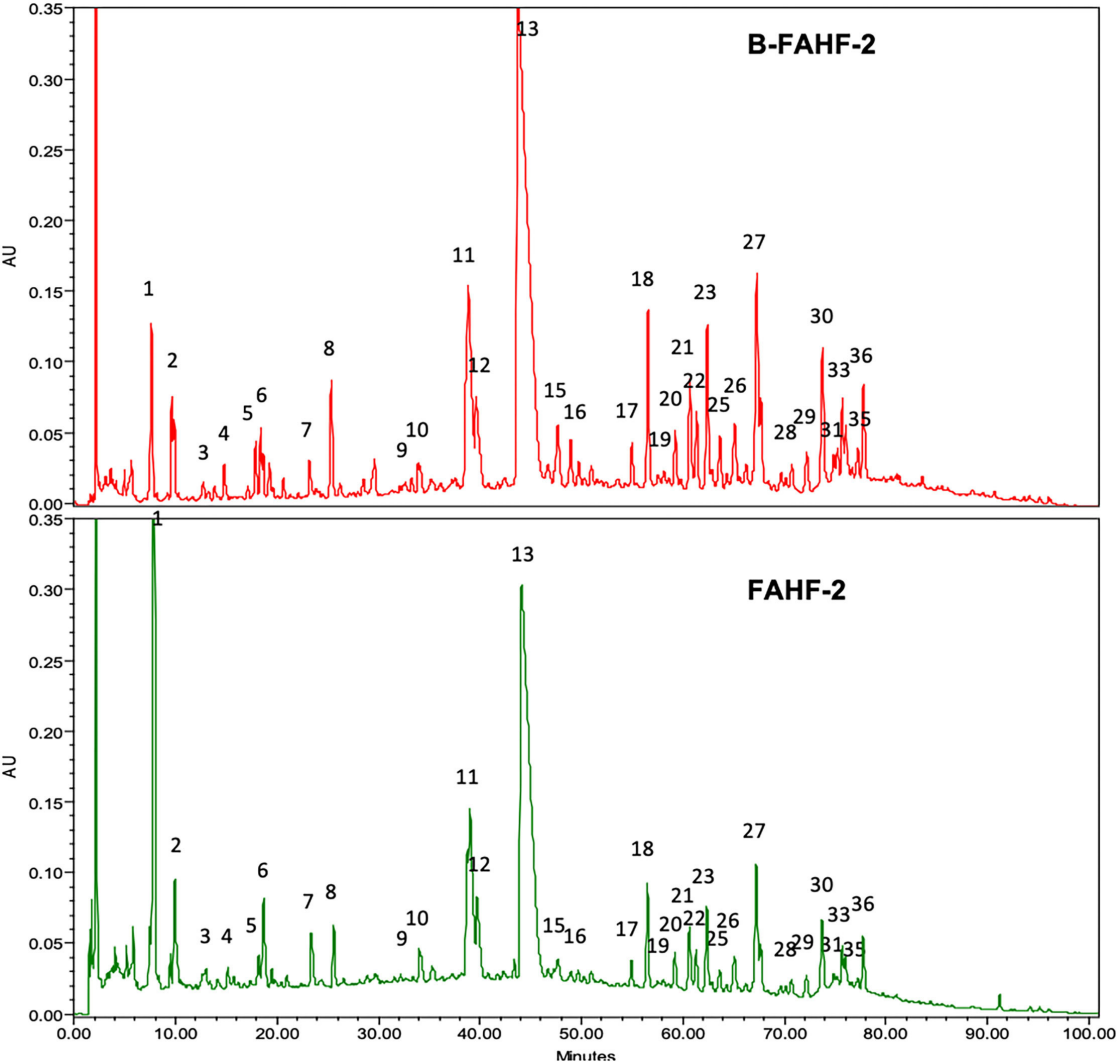
HPLC fingerprints of B-FAHF-2 (top) and FAHF-2 (bottom) demonstrating the presence of the same compounds. The HPLC major peaks in three key herbs of FAHF-2 and B-FAHF-2, Huang-Bai, Ling-Zhi and Wu-Mei overlap with FAHF-2 and B-FAHF-2 in on-line ultraviolet (UV) spectra and retention time (t_R_). Huang-Bai contributes to peaks 1, 5–8, 12–14. Ling-Zhi contributes to peaks 1–4, 7, 13, 16–19, 31, 32, 35, 36. Wu-Mei contributes to peak 1–6.

After butanol extraction of FAHF-2 was completed, B-FAHF-2 and FAHF-2 were examined by HPLC to ensure that the compounds were similar. HPLC fingerprints (Figure 1) demonstrate similar patterns with peaks at similar absorbance units and retention times, indicating that they contain the same compounds.

**Figure 2:**
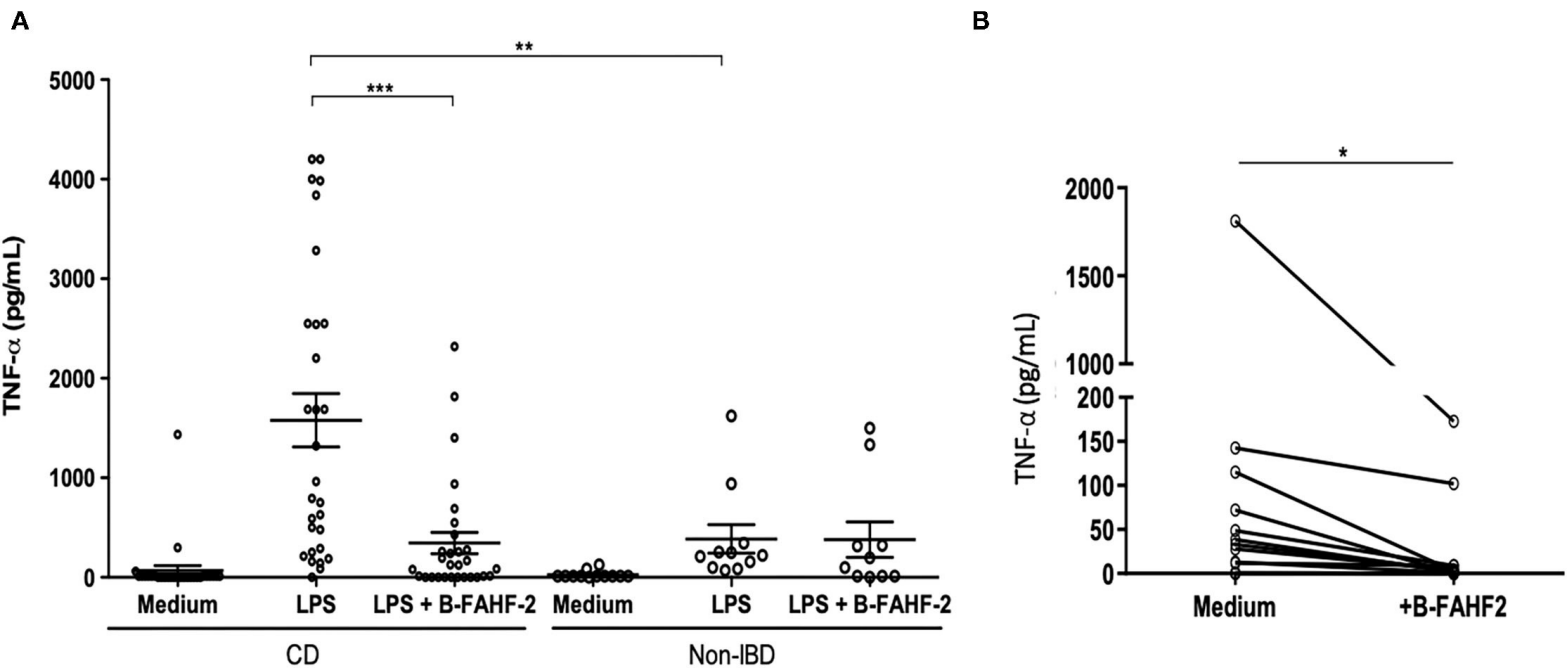
B-FAHF-2 suppressed production of TNF-α by PBMCs and by colonic mucosa from pediatric subjects with CD. **(A)** TNF-α levels from PBMCs after stimulation with LPS in CD and non-IBD controls and then after treatment with B-FAHF-2 (CD *n* = 29, Non-IBD *n* = 12). **(B)** TNF-α levels from colonic biopsies from pediatric subjects with CD cultured in medium or with the addition of B-FAHF-2 (*n* = 20) ^(***^p < 0.001, ^**^p < 0.01, ^*^p < 0.05).

FAHF-2 suppressed production of TNF-α IFN-γ, and IL-12 from PBMCs from pediatric subjects with CD (5) and therefore we examined B-FAHF-2's effects on TNF-α production as a representative cytokine. We first tested the effect of B-FAHF-2 (60 μg/ml) on TNF-α secretion by PBMCs from 29 CD subjects and 12 non-IBD controls (Table 2). PBMCs from CD subjects secreted significantly more TNF-α upon LPS stimulation as compared to non-IBD controls (*p* < 0.01) (Figure 2A). TNF-α levels were significantly reduced when CD PBMCs were cultured with B-FAHF-2 (*p* < 0.001) (Figure 2A). No cytotoxicity was detected (Data not shown).

To be effective at treating CD, B-FAHF-2 should have effects on intestinal mucosa and inflammatory cytokine secretion. FAHF-2 suppressed production of TNF-α, IL-1β, IL-6, and IL-17 from inflamed colonic mucosa from pediatric subjects with CD (5) and therefore we examined B-FAHF-2's effects on TNF-α production as a representative cytokine. We incubated inflamed colonic mucosa from subjects with CD with and without B-FAHF-2 and quantified TNF-α from the supernatant. B-FAHF-2 significantly suppressed the production of TNF-α from inflamed biopsies from subjects with CD (*p* < 0.05) (Figure 2B).

Thus, like FAHF-2, B-FAHF-2 significantly suppressed TNF-α production by PBMCs and inflamed colonic mucosa from pediatric subjects with CD.

### B-FAHF-2 Stimulated Increased Production of GM-CSF by PBMCs and Colonic Mucosa From Pediatric Subjects With CD

**Figure 3:**
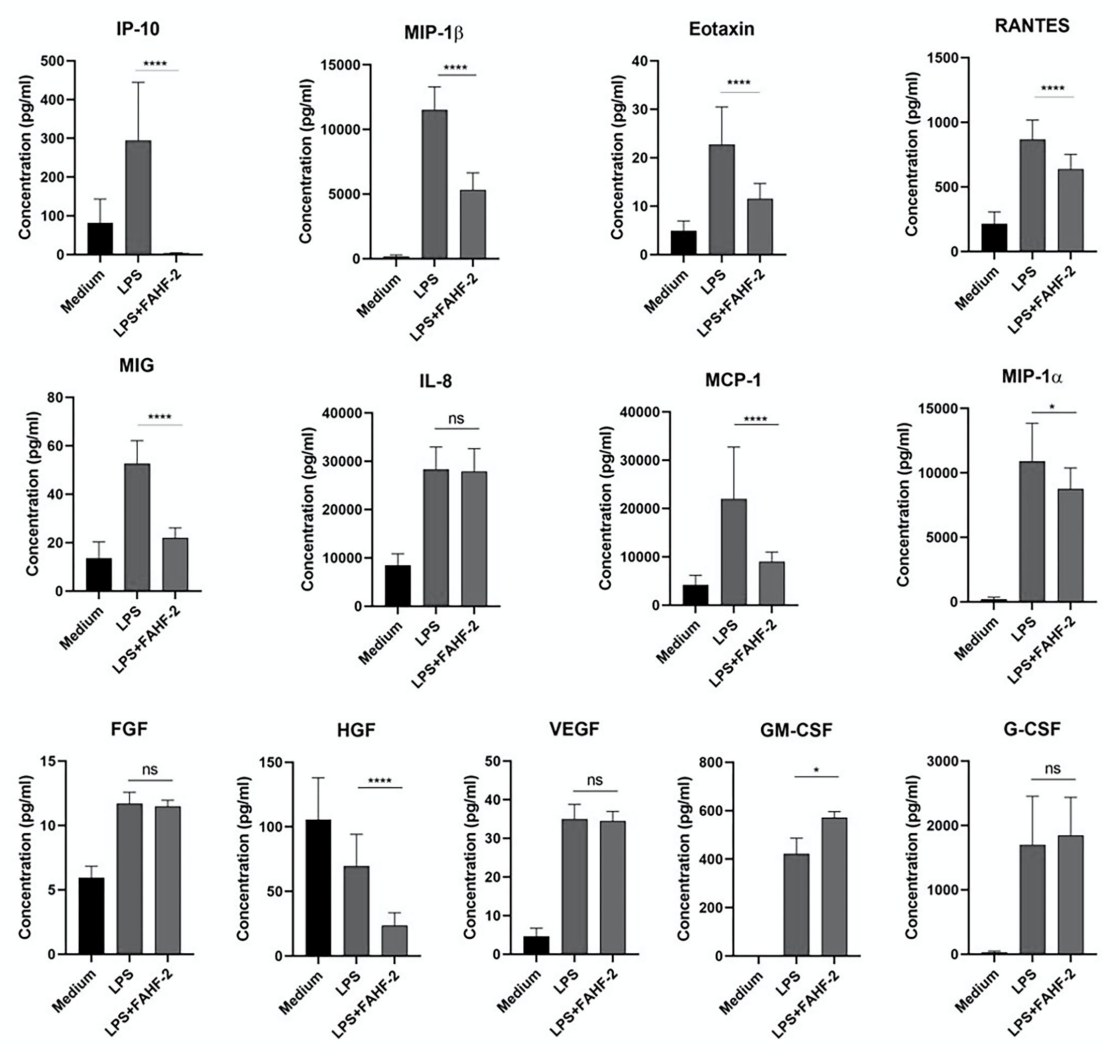
FAHF-2 modulated chemokine and growth factor production by PBMCs from pediatric CD subjects. Production of IP-10, MIP-1β, Eotaxin, RANTES, MIG, MCP-1, MIP-1α, HGF and GM-CSF by LPS- stimulated PBMCs from pediatric CD subjects was significantly altered when cultured with FAHF-2 (LPS+FAHF-2) versus without it (LPS) (*n* = 14, ns, not significant, ^*^p < 0.05, ^****^p < 0.0001).

FAHF-2 had an immunomodulatory effect that is best demonstrated by its stimulation of increased production of GM-CSF while at the same time suppressing production of multiple inflammatory chemokines (IP-10, MIP-1β, eotaxin, RANTES, MIG, MCP-1, and MIP-1α) and HGF from PBMCs from pediatric subjects with CD (Figure 3).

**Figure 4:**
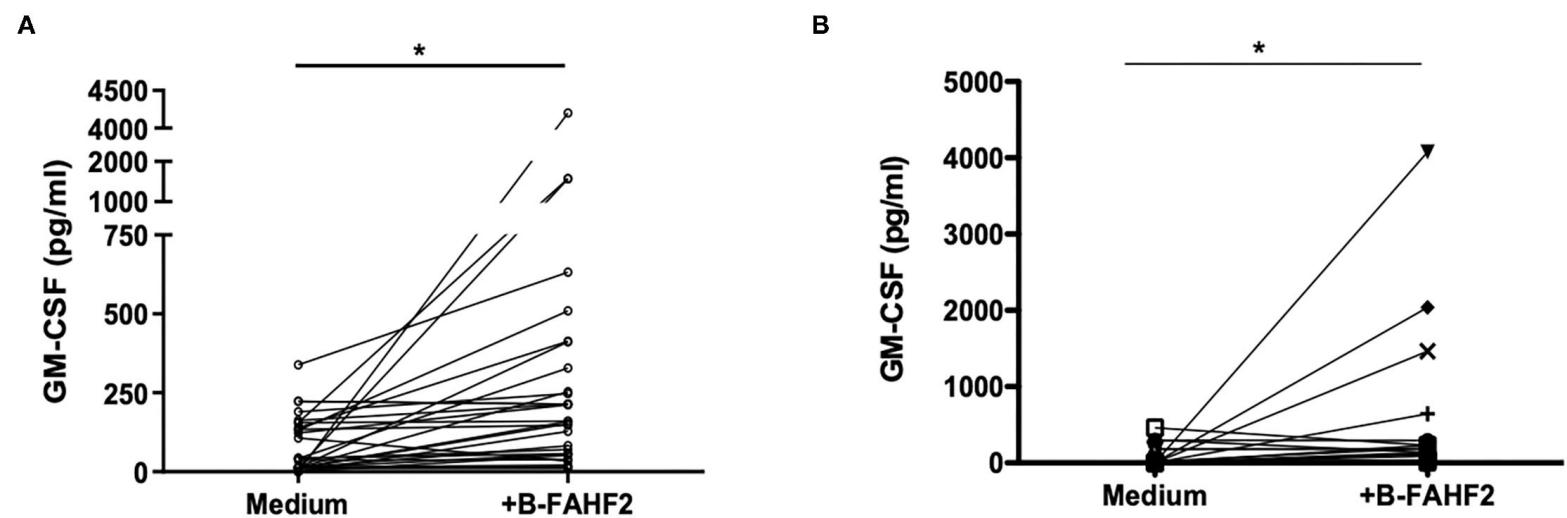
B-FAHF-2 stimulated increased production of GM-CSF by PBMCs and colonic mucosa from pediatric subjects with CD. **(A)** GM-CSF levels from PBMCs from pediatric subjects with CD cultured in medium or with the addition of B-FAHF-2 (*n* = 29). **(B)** GM-CSF levels from colonic biopsies from pediatric subjects with CD cultured in medium or with the addition of B-FAHF-2 (*n* = 20) (^*^p < 0.05).

Therefore, using GM-CSF as representative of the immunomodulatory capacity FAHF-2, we tested the effect of B-FAHF-2 on GM-CSF secretion by unstimulated PBMCs from all 29 pediatric subjects with CD. PBMCs cultured with B-FAHF-2 produced more GM-CSF than when cultured in medium alone (Figure 4A) (*p* < 0.05). B-FAHF-2 also stimulated significantly increased production of GM-CSF from biopsies from pediatric subjects with CD (Figure 4B) (*p* < 0.05).

### B-FAHF-2 Increased GM-CSF Production by PBMCs From Pediatric Subjects With CD Who Have GM-CSF Antibodies

**Figure 5:**
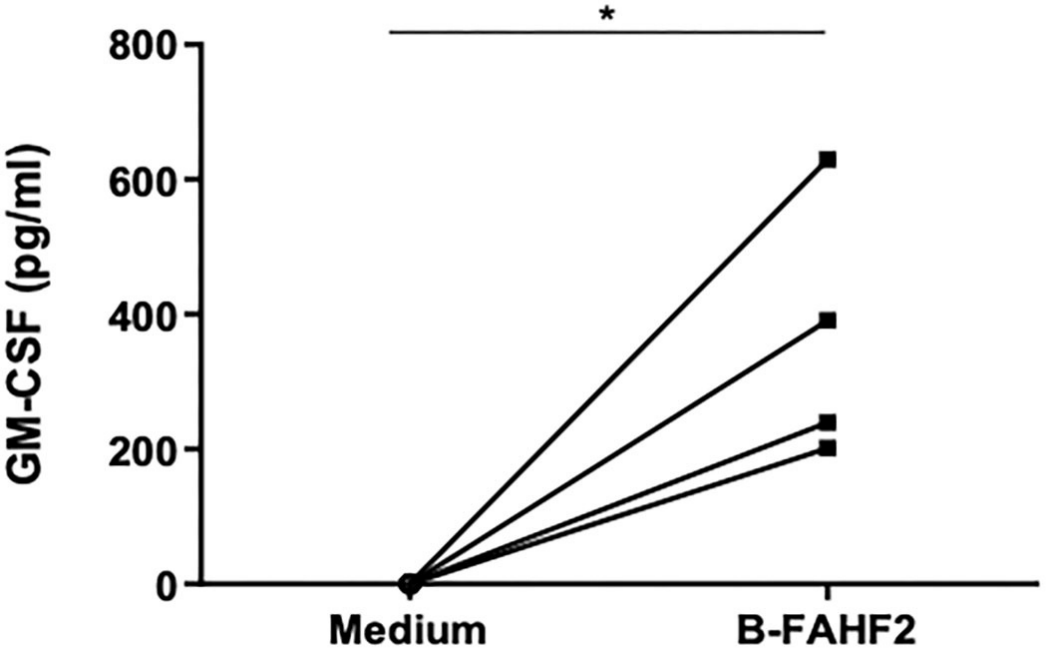
B-FAHF-2 caused increased GM-CSF production by PBMCs from pediatric subjects with CD who have GM-CSF antibodies. GM-CSF levels from PBMCs from four pediatric subjects with CD and GM-CSF antibodies cultured in medium alone or with the addition of B-FAHF-2 (*n* = 4, ^*^p < 0.05).

Given the effect of B-FAHF-2 on GM-CSF and the possibility that it might be beneficial in a subset of patients with GM-CSF antibodies, we tested the serum from all subjects. We found that four of the 29 subjects with CD had GM-CSF antibodies. B-FAHF-2 significantly increased GM-CSF production by PBMCs from these 4 subjects (*p* < 0.05) (Figure 5) despite them having antibodies to GM-CSF.

### Only B-FAHF-2 and Huang Bai Were Effective at Both Suppressing TNF-α Production and Inducing GM-CSF Production by PBMCs From Pediatric Subjects With CD

**Figure 6:**
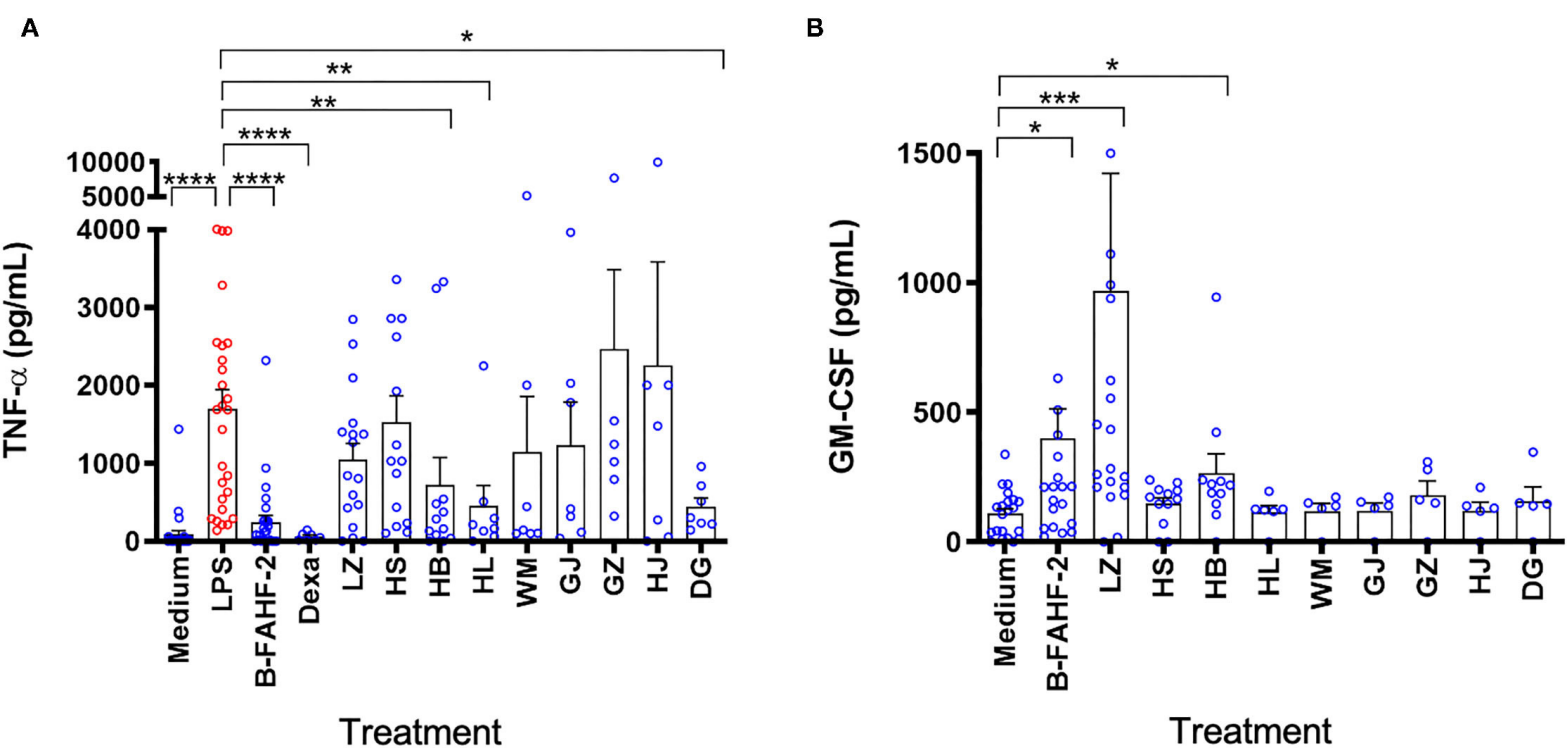
Only B-FAHF-2 and Huang Bai suppressed TNF-α and induced GM-CSF production by PBMCs from pediatric subjects with CD. **(A)** TNF-α levels from PBMCs from pediatric subjects with CD stimulated with LPS and treated with B-FAHF-2, dexamethasone (Dexa), *Ganoderma lucidum/*Ling Zhi (LZ), *Panax ginseng/*Hong Shen (HS), *Phellodendron chinense/*Huang Bai (HB), *Coptis chinensis/*Huang Lian (HL), *Prunus mume/Wu Mei (WM), Zingiber officinalis/*Gan Jiang (GJ), *Cinnamomum cassia/*Gui Zhi (GZ), *Zanthoxylum bungeanum/*Chuan Jiao (HJ), or *Angelica sinensis/*Dang Gui (DG). **(B)** GM-CSF levels from PBMCs from pediatric subjects with CD treated with B-FAHF-2, dexamethasone (Dexa), *Ganoderma lucidum/* Ling Zhi (LZ), *Panax ginseng/* Hong Shen (HS), *Phellodendron chinense/*Huang Bai (HB), *Coptis chinensis/*Huang Lian (HL), *Prunus mume/Wu Mei (WM), Zingiber officinalis/*Gan Jiang (GJ), *Cinnamomum cassia/*Gui Zhi (GZ), *Zanthoxylum bungeanum/*Chuan Jiao (HJ), or *Angelica sinensis/*Dang Gui (DG) (*n* 7–28) (^****^p < 0.0001, ^***^p < 0.001, ^**^p < 0.01, ^*^p < 0.05 as compared with LPS stimulated PBMCs for TNF-α or as compared to medium alone for GM-CSF).

Given the potent suppressive effect of FAHF-2 and B-FAHF-2 on TNF-α production, (5) each herb that comprises them [*Prunus mume (Wu Mei), Zanthoxylum bungeanum* (Chuan Jiao), *Angelica sinensis* (Dang Gui), *Zingiber officinalis* (Gan Jiang), *Cinnamomum cassia* (Gui Zhi), *Phellodendron chinense* (Huang Bai), *Coptis chinensis* (Huang Lian), *Panax ginseng* (Hong Shen) and *Ganoderma lucidum* (Ling Zhi)] was tested for the ability to suppress production of TNF-α from LPS stimulated PBMCs from pediatric subjects with CD. LPS caused a marked increase in production of TNF-α. B-FAHF-2, Dexamethasone (Dexa), Huang Bai (HB), Huang Lian (HL) and Dang Gui (DG) significantly reduced production of TNF-α (*p* < 0.0001, *p* < 0.001, *p* < 0.01, *p* < 0.05, and *p* < 0.05 respectively) (Figure 6A).

We then tested the effect of B-FAHF-2 and each herb that comprises it on the production of GM-CSF. B-FAHF-2, Ling Zhi (LZ) and Huang Bai (HB) induced significantly increased production of GM-CSF by unstimulated PBMCs from pediatric subjects with CD (*p* < 0.05, *p* < 0.001, and *p* < 0.05, respectively) (Figure 6B).

Overall, only B-FAHF-2 and Huang Bai (HB) could both decrease TNF-α and increase GM-CSF production by PBMCs unlike any of the other individual herbs.

### B-FAHF-2 Alleviated Colitis in a Murine Model

**Figure 7:**
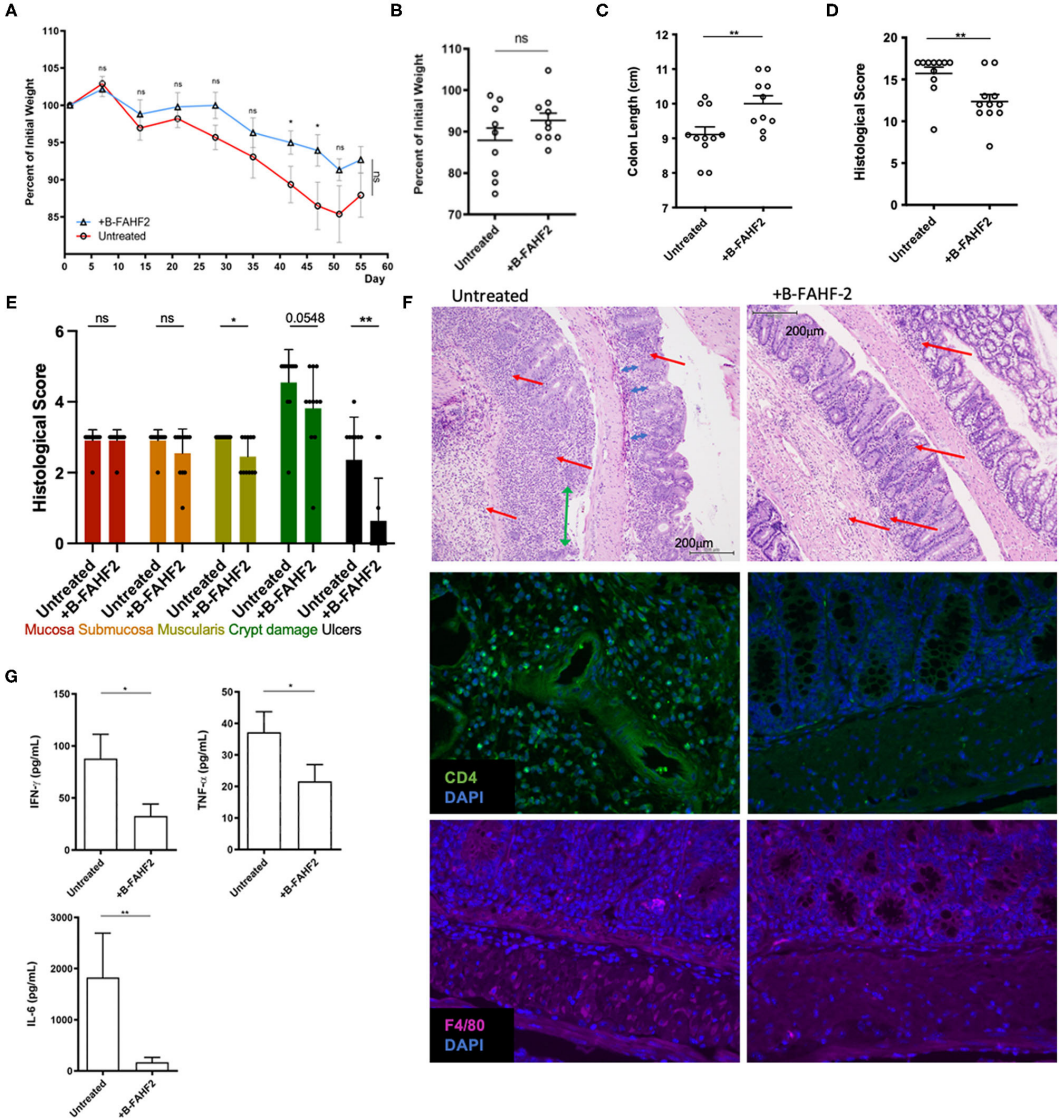
B-FAHF-2 alleviated colitis in the CD45RB^hi^ transfer model. **(A)** Weight curves of mice treated (+B-FAHF-2) or untreated with B-FAHF-2. **(B)** The final percentage of initial body weight as measured at the time the mice were sacrificed. **(C)** Colon length of mice treated (+B-FAHF-2) or untreated with B-FAHF-2. **(D)** The total histological scores of inflammation of the colon of mice treated (+B-FAHF-2) or untreated. **(E)** The histological sub-scores for inflammation in the mucosa, submucosa, and muscularis as well as for crypt damage and the extent of ulceration in mice treated (+B-FAHF-2) or untreated. **(F)** A representative H & E stained histological figure demonstrating the untreated specimen with marked inflammatory infiltrate including neutrophils (red arrow), loss of crypts (blue arrow), crypt distortion, and ulceration (green arrow) of the mucosa vs. the B-FAHF-2 treated specimen which has significantly less inflammatory infiltrate and disordered crypts but no ulceration and minimal loss of crypts. Immunofluorescence staining for CD4^+^ T cells and macrophages (F4/80) in untreated and +B-FAHF-2 groups. **(G)** Cytokine production by colonic mucosa from mice treated (+B-FAHF-2) or untreated with B-FAHF-2 (*n* = 10 per group, ns, not significant, ^*^p < 0.05, ^**^p < 0.01).

In the CD45RB^hi^ T cell transfer model of colitis, treatment with FAHF-2 decreased colitis progression as evidenced by decreased weight loss, histological inflammation and production of TNF-α, IFN-γ, IL-6, and IL-17 from the colon (5). Therefore, we assessed the effect of B-FAHF-2 *in vivo* using the CD45RB^hi^ T cell transfer model of colitis, which exhibits features like those found in CD including transmural colitis and elevated levels of TNF-α. B-FAHF-2 treated mice trended toward less weight loss (Figures 7A,B) and had significantly less colon shortening than untreated controls (*p* < 0.05) (Figure 7C). The B-FAHF-2 treated mice had significantly less histological inflammation with less muscularis inflammatory infiltrates, crypt damage, and ulceration (*p* < 0.05, *p* < 0.05, *p* = 0.0548, and *p* < 0.01, respectively) (Figures 7D–F). The scoring system used takes into account changes in neutrophil infiltrates. Thus, we also looked by IF at CD4^+^ T cell and macrophage infiltrates and found that both were decreased in the B-FAHF-2 treated group (Figure 7F). Inflammatory cytokine production by colonic tissue in B-FAHF-2 treated mice was reduced: TNF-α, IFN-γ, and IL-6 (*p* < 0.05, *p* < 0.05, *p* < 0.01, respectively) (Figure 7G). Levels of IL-17 and IL-10 were not detectable in both groups (data not shown). Finally, to examine if B-FAHF-2 affects regulatory T cells, we examined TGF- β1, TGF-β2, Foxp3, and IL-10 by RT-PCR and found no significant differences between the groups (data not shown).

Thus, B-FAHF-2 alleviates colitis and inhibits the inflammatory milieu associated with colitis.

## Discussion

In this continuation of our studies on FAHF-2 as a potential treatment for CD, we first sought to lessen the pill burden of FAHF-2 required for human trials by purifying the active compounds and then by examining the individual herbs. Our data demonstrates that the purified product, B-FAHF-2, was effective at suppression of TNF-α production by both PBMCs and mucosal biopsies from pediatric subjects with CD at 20% of the dose of FAHF-2. In a murine model, B-FAHF-2 was effective at alleviating colitis at this same dose. B-FAHF-2 also caused an increase in GM-CSF production even in those subjects with antibodies against it. Of the individual herbs within FAHF-2 and B-FAHF-2, only Huang Bai was effective at both suppressing TNF-α and stimulating GM-CSF production.

Purification of FAHF-2 using butanol extraction did not affect the ability of B-FAHF-2 to suppress TNF-α production *ex vivo* from human PBMCs and colonic mucosa nor its ability to abrogate colitis in a murine model. The components removed by butanol purification were non-medicinal components including fiber, wax, proteins and starch. Given that B-FAHF-2 has a similar HPLC fingerprint as FAHF-2, and similar effects on TNF-α suppression (5), B-FAHF-2 likely has similar immunomodulatory effects beyond what we have shown here. Importantly, the *in-vitro* dose of FAHF-2 that inhibited 60–70% of TNF-α production was 700 μg/mL whereas that of B-FAHF-2 is only 60 μg/mL. In human trials this equates to a pill burden reduction from 36 pills daily for FAHF-2 to 10 pills maximum per day for B-FAHF-2 (6). This reduction would allow clinical trials to proceed with better participant compliance. An even more purified version of B-FAHF-2 is currently in a phase II trial as an adjunct therapy for children and adults with multiple food allergies (24), and a clinical trial in patients with mild-to-moderate CD, where there is a lack of FDA approved medications, is about to begin (25).

Our results on the effect of FAHF-2 on chemokines and growth factors showed that the formulation is immunomodulatory and not broadly immunosuppressant. Studies have shown that alterations of GM-CSF function may be involved in a subset of IBD cases (14). A recombinant human form of GM-CSF, sargramostim, had positive effects in a pilot study of patients with CD but larger trials did not show improved clinical outcomes (26, 27). This is likely due to heterogeneity in pathogenic mechanisms. Therefore, GM-CSF may be an appropriate therapy for a subgroup of CD patients which the larger clinical trial did not target specifically. We demonstrate for the first time, that B-FAHF-2 increases production of GM-CSF from PBMCs and from colonic mucosa from children with CD. The most likely therapeutic target for this increase would be a subset of CD patients who have been shown to have antibodies against GM-CSF and thus low levels of GM-CSF. In our study population, only 4 children had significant antibodies against GM-CSF and, independent of the presence of antibodies, B-FAHF-2 significantly increased production of GM-CSF by PBMCs from these subjects.

The complexity of Traditional Chinese Medicine makes these therapies particularly difficult to study. Each herbal formulation including B-FAHF-2, incorporates multiple herbs, which in turn are made up of numerous chemicals. The active components of these complex mixtures work synergistically and removal of some components, even if inactive, can destabilize the active components (28). In addition, many of the components may be transformed *in vivo* into more complex active metabolites that could be eliminated unintentionally. Thus, we were not surprised that only one of the individual herbs, Huang Bai, could both suppress TNF-α and stimulate GM-CSF production although not with the same effect as B-FAHF-2. Other individual herbs were able to either suppress TNF-α or increase GM-CSF production but none did both. Interestingly, our group has attempted to isolate active compounds within this complex formulation but has not shown that individual compounds or smaller combinations of them have the same effects as the entire mixture together. In addition to the complexity of the formulation which makes it difficult to study, further challenges to performing clinical trials include obtaining reliable sources for the raw herbal components, ensuring no pesticide or heavy metal contamination, and confirming consistency of the chemical makeup of each batch of herbal medicine. Finally, herbal formulas have often been found to have hepatotoxicity. FAHF-2 and B-FAHF-2 were made with the removal of potentially hepatoxic herbs and data in prior murine and human studies have shown no hepatoxicity as evidenced by this formulation receiving an Investigational New Drug status from the FDA (8, 29).

Our study is the next step toward the development of this formulation into an herbal therapy for CD but additional refinement of the formulation and further larger studies must be performed. This study has several other limitations. We used TNF-α and GM-CSF as surrogate markers for the immunomodulatory capabilities of B-FAHF-2 but examination of a multitude of cytokines, chemokines and growth factors should be examined as was done with FAHF-2. In prior studies we examined the mechanism of how FAHF-2 suppressed inflammatory cytokine production and found that the NFκB pathway was inhibited. This is unlikely to explain the mechanism causing increases in GM-CSF production and will need to be investigated in future studies. In addition, we had only a small sample of subjects with antibodies against GM-CSF. The effects of B-FAHF-2 in this sub-group should be confirmed in larger patient populations.

## Conclusions

In conclusion, B-FAHF-2 suppresses production of TNF-α, a major inflammatory cytokine involved in the pathogenesis of CD, to a similar extent as FAHF-2 but at a lower dose. B-FAHF-2 warrants further clinical investigation to determine its efficacy in CD. B-FAHF-2 also increased production of GM-CSF and thus warrants further investigation in a sub-group of CD patients who have low levels of or antibodies against GM-CSF.

## Data Availability Statement

The raw data supporting the conclusions of this article will be made available by the authors, without undue reservation.

## Ethics Statement

The studies involving human participants were reviewed and approved by Icahn School of Medicine at Mount Sinai Institutional Review Board. Written informed consent to participate in this study was provided by the participants' legal guardian/next of kin. The animal study was reviewed and approved by Icahn School of Medicine at Mount Sinai Institutional Animal Care and Use Committee.

## Author Contributions

XC and JL: validation, formal analysis, writing—original draft, and visualization. YS and SG: methodology, formal analysis, and writing—review and editing. NY: resources, writing—review, and editing. VG, WH, and EC: formal analysis and writing—review and editing. NP, JJ, and KB: specimen collection and writing—review and editing. MD: conceptualization, writing—review and editing. X-ML: conceptualization, writing—review and editing, supervision, project administration, and funding acquisition. DD: conceptualization, formal analysis, resources, writing—review and editing, visualization, supervision, project administration, and funding acquisition. All authors contributed to the article and approved the submitted version.

## Funding

This work was partially supported by National Institute of Diabetes Digestive and Kidney Diseases, R03 DK117218 and The Leona M. and Harry B. Helmsley Charitable Trust (to DD), U01 DK1241654 (to SG), and partially supported by NIH 2R01 AT001495-01A1 (to X-ML).

## Conflict of Interest

X-ML formerly had patents with Herbal Springs for FAHF-2 for treating food allergy. SG is a named inventor of a patent application related to GM-CSF autoantibodies in immune-related colitis. This is filed through the Icahn School of Medicine at Mount Sinai and is currently unlicensed. SG reports consultancy and/or advisory roles for Merck and OncoMed, and research funding from Bristol-Myers Squibb, Celgene, Genentech, Immune Design, Janssen R&D, Pfizer, Regeneron, and Takeda. The remaining authors declare that the research was conducted in the absence of any commercial or financial relationships that could be construed as a potential conflict of interest.

## Publisher's Note

All claims expressed in this article are solely those of the authors and do not necessarily represent those of their affiliated organizations, or those of the publisher, the editors and the reviewers. Any product that may be evaluated in this article, or claim that may be made by its manufacturer, is not guaranteed or endorsed by the publisher.

## Abbreviations

FAHF-2: Food Allergy Herbal Formula-2
B-FAHF-2: butanol purified FAHF-2
IBD: inflammatory bowel disease
CD: Crohn's disease
HPLC: high pressure liquid chromatography
PBMCs: Peripheral blood mononuclear cells.
